# Supplementary material for: Effect of Electrical Conductivity of Nutrient Solution and Light Spectra on the Main Phytochemical Content of Sonchus tenerrimus L. Under Wild and Controlled Environments
Source: Plants (Basel). 2025 Sep 8;14(17):2811. doi: 10.3390/plants14172811 (PMC12431034; doi:10.3390/plants14172811)
Supplement: Supplementary file 1 [file plants-14-02811-s001.zip › plants-3821950-supplementary.pdf]

Supplementary Materials of the article:

Effect of Electrical Conductivity of Nutrient Solution and Light Spectra on the Main Phytochemical Content of *Sonchus tenerrimus* L. Under Wild and Controlled Environments

Supplementary File S1

Material and Methods

1. Reagents and chemicals

Unless stated otherwise, all chemicals and solvents were procured from Merck (Madrid, Spain). L-ascorbic acid was procured from Labkem (Barcelona, Spain). Aluminum chloride and sodium carbonate were obtained from Sigma-Aldrich Co. (St Louis, MO, USA). Sodium nitrite, sodium hydroxide, and oxalic acid were acquired from Panreac (Barcelona, Spain). The Folin–Ciocâlteu (F-C) reagent, β-carotene, DPPH, $ABTS^{+\bullet}$, and Trolox standards were obtained from Sigma-Aldrich Co. (St Louis, MO, USA). Water purification was carried out using a Milli-Q system (Millipore). All chemicals and solvents utilized in this study were of analytical grade.

2. Growth, fertigation, and lighting conditions applied to cultivated Sonchus tenerrimus

S. tenerrimus plants were grown under two independent experiments. The first one used three treatments with different electrical conductivities in the nutrient solution: 1.2 (C1), 1.8 (C2), 2.4 (C3), and 3.0 (C4) dS m^{-1}. A Roblan® L18 T8 white LED lamp (Toledo, Spain) was used for the test. In the second experiment, four LED lamps were evaluated: L18 NS1, L18 AP67, and L18 NS12 Valoya® (Helsinki, Finland) as treatment 1, 2, and 3, respectively (L1, L2, and L3), and L18 T8 Roblan® as control (L4).

For both experiments, three light-emitting diode (LED) lamps were used, all on a surface of 0.504 m^2. All lamps were the same length and power (18 W). The spectra of each treatment were measured with a UPRtek MK350S LED (UPRtek, Taiwan). LP471-PHOT and LP471-PAR sensors (Delta OHM®, Padua, Italy) were used to measure luminance (lux) and photosynthetic photon flux, PPF (µmol m^{-2} s^{-1}), as described by Ferrón-Carrillo et al. [10].

The application of the fertigation was performed when the water in the culture unit reached 10% of the readily available water, and the necessary volume was added to obtain between 15 and 25% drainage [19]. For each treatment, two controls were established as fertigation controls, consisting of a control dripper and a drainage tray that served as measurement and monitoring points for the fertigation supplied and the uptake response. The volume, pH, electrical conductivity (EC), nitrate and potassium content of the supplied nutrient solution, and drainage were measured daily in each pot with a container that was adapted to its morphology. The pH and EC were monitored using systems from HORIBA Ltd. (LAQUAact PC110-K). The contents of nitrate and potassium were measured with systems from HORIBA Ltd. Japan (LAQUAtwin B-741 and LAQUAtwin B-731, respectively). The absorption of nitrates and potassium in mmol·plant^{-1} was quantified based on the balance between input and output [19]. Volume (L) was measured using a test tube graduated to the hundredth of a millimeter.

To perform the correlations between the determinant parameters with the rest of the phytochemical parameters in each experiment, the EC was considered in the salinity essay and in the illumination essay ratio red/far red. The EC of the substrate and of the soil where the plants were growing were measured using saturated extract techniques, while the red/far red ratio was measured as described in Nájera and Urrestarazu [31].

3. Evaluation of growth parameters

The evaluation of growth parameters was carried out 30 days after transplantation. The experimental unit was four plants per treatment and four replicates of each treatment. The plants were divided by their different organs; the fresh weight of roots, stems, and leaves was obtained, then the dry weight was obtained by placing the material in an oven (Thermo Scientific Heratherm, Germany) at 85 °C until achieving a constant weight. A precision analytical

balance (Adventurer Analytical OHAUS Model AX 124/E, USA) was used, expressing the result as g plant^{-1}.

After harvesting, plants were stored in thermal bags and frozen at -24 ºC until processing. Once in the laboratory, the greens were labeled, weighed, measured, and placed in a glass desiccator until analysis.

References

Ferrón-Carrillo, F.; Cunha-Chiamolera, T.P.L.; Urrestarazu, M. Effect of ammonium nitrogen on pepper grown under soilless culture. *J. Plant Nutr.* **2022**, *45*, 113-122. https://doi.org/10.1080/01904167.2021.1943438

Nájera, C.; Urrestarazu, M. Effect of the intensity and spectral quality of LED light on yield and nitrate accumulation in vegetables. *HortScience* **2019**, *54*, 1745-1750. https://doi.org/10.21273/HORTSCI14263-19

Peçanha, D.A.; Peña J.A.M.; Freitas, M.S.M.; Chourak, Y.; Urrestarazu, M. Effect of light spectra on stem cutting rooting and lavender growth. *Acta Sci. Agron.* **2023**, *45*, e58864. https://doi.org/10.4025/actasciagron.v45i1.58864

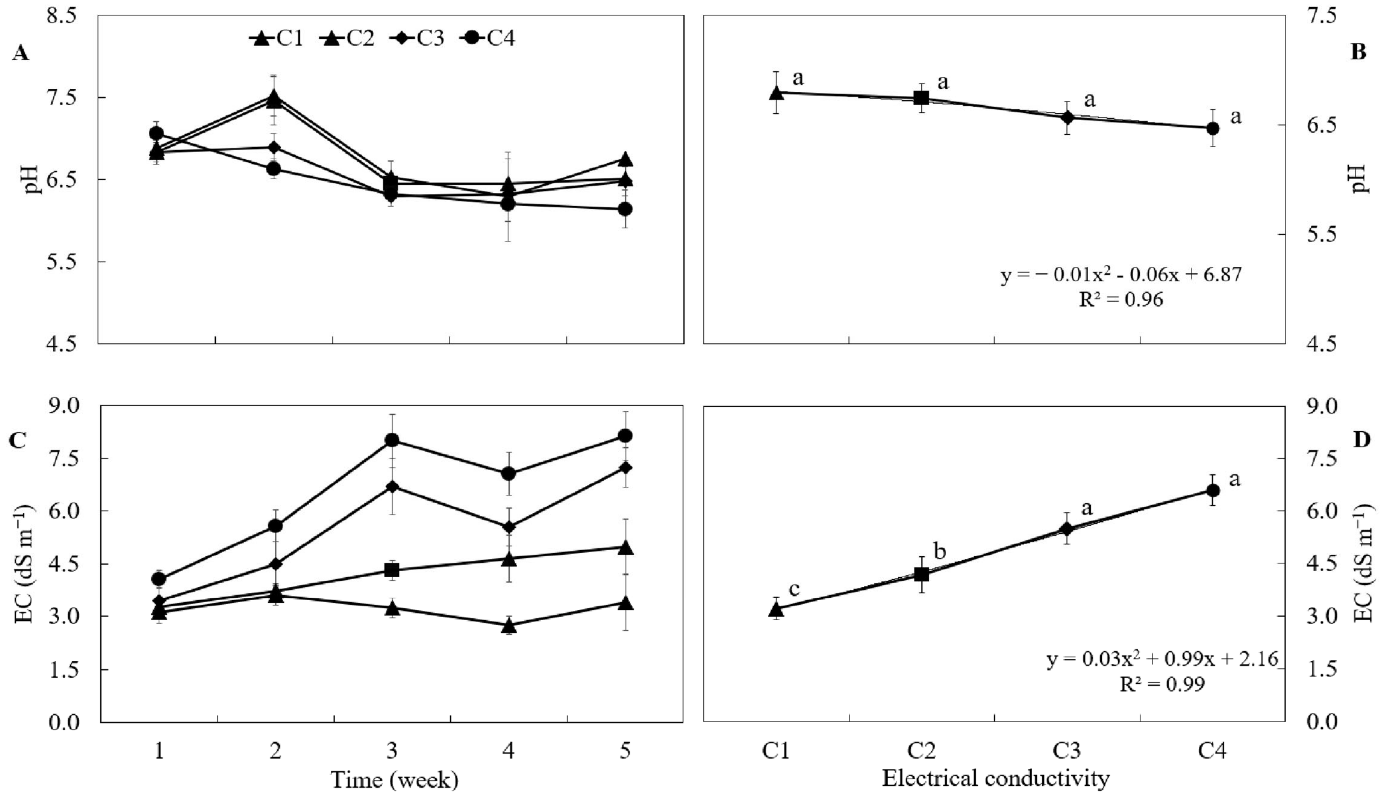

Supplementary Figure S1. pH (A and B) and electrical conductivity (EC – C and D) of drainage in response to different nutrient solution EC levels in *S. tenerrimus* soilless culture. Treatments: C1, C2, C3, and C4 correspond to 1.2, 1.8, 2.4, and 3.0 $dS\,m^{-1}$, respectively. Different letters indicate statistically significant differences ($p < 0.05$) according to Tukey's test.

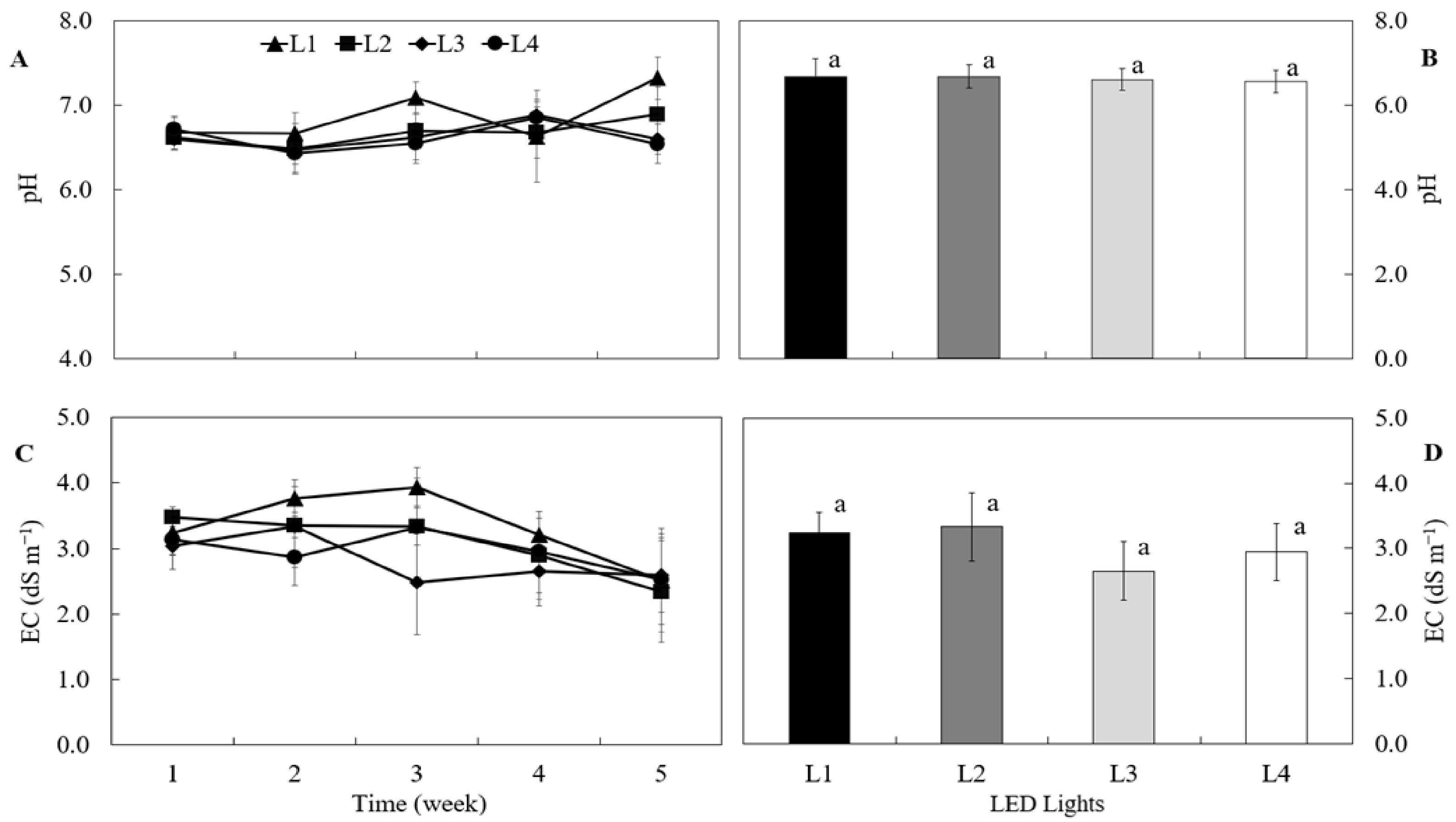

Supplementary Figure S2. pH (A and B) and electrical conductivity (EC – C and D) of drainage in response to different light spectra in *S. tenerrimus* soilless culture. Illumination treatments: L1 – L18 NS1 Valoya®; L2 – L18 AP67 Valoya®; L3 – L18 NS12 Valoya®; L4 – L18 T8 Roblan®. Different letters indicate statistically significant differences ($p < 0.05$) according to Tukey's test.

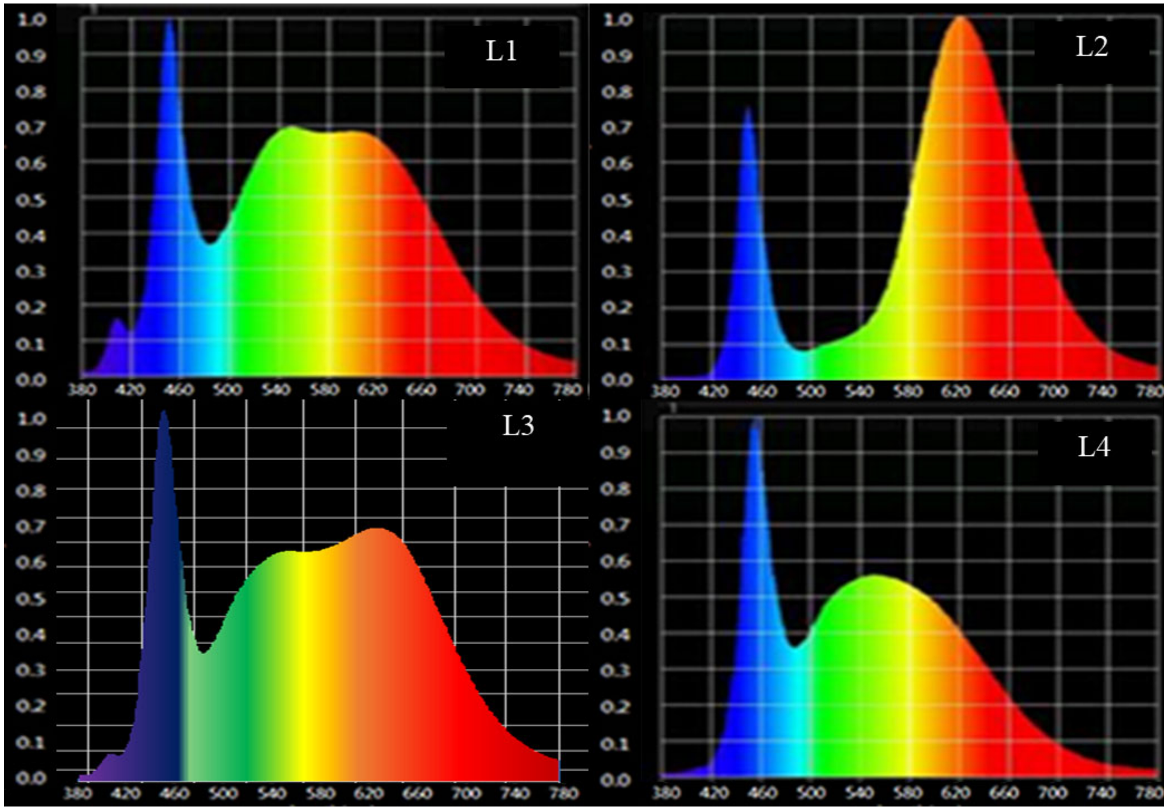

Supplementary Figure S3. Light spectra of the LED lamps used as treatments during the crop of *S. tenerrimus* plants. Treatments: L1 – L18 NS1 Valoya®; L2 – L18 AP67 Valoya®; L3 – L18 NS12 Valoya®; L4 – L18 T8 Roblan®.

Supplementary Table S1. Composition of the nutrient solutions used for culturing *Sonchus tenerrimus* plants[a.]

Electrical conductivity (dS m^{-1})	pH	Macronutrients (mM)						Micronutrients (μM)					
		NO_3^-	$H_2PO_4^-$	SO_4^{2-}	K^+	Ca^{2+}	Mg^{2+}	Fe	Mn	Cu	Zn	B	Mo
1.2	5.8	6.65	1.40	1.26	2.86	3.00	0.91	15	10	0.75	5	30	0.5
1.8	5.8	9.99	2.12	1.96	4.28	4.50	1.37	15	10	0.75	5	30	0.5
2.4	5.8	13.31	2.82	2.61	5.71	6.00	1.82	15	10	0.75	5	30	0.5
3.0	5.8	16.54	3.42	3.17	7.14	7.50	2.28	15	10	0.75	5	30	0.5

[a] Based on Sonneveld, and Straver, [72].
